# Evaluation of the clinical value of carbon nanoparticles in endoscopic thyroidectomy and prophylactic central neck dissection through total mammary areolas approach for thyroid cancer

**DOI:** 10.1186/s12957-021-02427-8

**Published:** 2021-11-04

**Authors:** Jie He, Chaojie Zhang, Zeyu Zhang, Fada Xia

**Affiliations:** 1 Department of Breast and Thyroid Surgery, Hunan Provincial People’s Hospital (First Affiliated Hospital of Hunan Normal University), Changsha, 410005 Hunan China; 2 Department of General Surgery, Xiangya Hospital, Central South University, No. 87 Xiangya Road, Changsha, 410008 China

**Keywords:** Endoscopic thyroidectomy, Prophylactic central neck dissection, Carbon nanoparticles

## Abstract

**Background:**

Carbon nanoparticles (CNs) are tracers used in thyroid surgery of patients with thyroid cancer (TC) to help remove lymph nodes and protect the parathyroid gland. The facilitative effect of carbon nanoparticles in endoscopic thyroidectomy and prophylactic central neck dissection (pCND) has not been reported.

**Methods:**

The protective effect on parathyroid gland (PG) function and the numbers of identified parathyroid glands and central lymph nodes in endoscopic thyroid surgery through the total mammary areolas approach were compared between the CN and control groups.

**Results:**

All endoscopic thyroidectomies were successfully completed. No difference was found in either group regarding the general characteristics or operative complications. The mean number of superior PGs and inferior PGs identified in situ or in the dissected central lymph tissues was not different between the groups. The mean number of lymph nodes removed by unilateral CND was greater in the CN group than in the control group. However, there was no difference in the number of harvested lymph nodes when excluding the LNs less than 5 mm, which exhibit an extremely low metastatic rate.

**Conclusion:**

Carbon nanoparticles do not improve the protective effect on the parathyroid gland, especially the inferior glands, in endoscopic thyroid surgery through the total mammary areolas approach. There is no need to use CNs to facilitate the lymph node harvest in endoscopic prophylactic unilateral CND.

## Introduction

Thyroid cancer (TC) is the fifth most common cancer in women [1, 2]. Among all of the types of TC, differentiated thyroid cancer is the most common and surgery is the standard therapy for the majority of patients [1]. During the past decades, thyroidectomy has been achieved through an anterior neck incision, which usually heals well with an acceptable cosmetic outcome. However, one-fifth of patients who undergo thyroidectomy have unsatisfactory experiences with neck scarring, and some of them even require plastic surgery. Thus, minimally invasive procedures have been invented to improve the cosmetic effect [3, 4]. To date, total endoscopic and robotic procedures have been gradually accepted for thyroidectomy [3]. However, controversies remain concerning the optimal surgical extension of TC using endoscopic approaches, such as the complications.

Intraoperative neuromonitoring is frequently used during thyroidectomy, especially in endoscopic approaches, to reduce the injury to the recurrent laryngeal nerve [5]. Indocyanine green and carbon nanoparticles (CNs) are tracers in current use to help remove central lymph nodes (CLNs) and protect the parathyroid glands (PGs) [6]. Carbon nanoparticles (CNs), with an average diameter of 150 nm, are larger than blood capillaries (20–50 nm) but smaller than lymphatic vessels (120–500 nm). CNs can only enter lymphatic vessels, where they are swallowed by macrophages and transported to the draining lymph node. The thyroid gland and draining lymph nodes are stained black, leaving the parathyroid glands unstained, enabling easy identification of parathyroid glands from surrounding tissues during conventional open thyroid surgeries [7, 8]. However, a consensus about CN usage has not yet been established regarding its improvement in the extent of neck dissection and the preservation of the parathyroid glands after TC surgery with endoscopic approaches [9, 10]. Endoscopic approaches are usually recommended during the treatment of early-stage cases. While the therapeutic central neck dissection (CND) is recommended among patients with lymph node invasion, prophylactic central neck dissection (pCND) remains highly controversial among patients without preoperative evidence of lymph node invasion (cN0). Certain studies have confirmed the effectiveness of pCND in preventing tumor recurrence; on the other hand, other studies also reported the opposite with an increased incidence of complications [11, 12]. The facilitative effect of carbon nanoparticles in protecting the parathyroid glands, especially the inferior glands, and in harvesting the central lymph nodes has not been reported for endoscopic thyroidectomy with pCND. We conducted this study to investigate the value of CNs in protecting parathyroid gland function, identifying the intraoperative parathyroid glands, and harvesting the central lymph nodes during endoscopic thyroid surgery.

## Materials and methods

### Patient enrollment

TC patients who underwent endoscopic surgery from January 2018 to December 2020 in Xiangya Hospital, Central South University, were enrolled retrospectively. The inclusion criteria were as follows: (1) Thyroid nodes were diagnosed as papillary thyroid cancer (PTC) by ultrasound-guided fine needle aspiration (FNA) before surgery and pathologically confirmed in paraffin-embedded tissues after surgery. (2) Total thyroidectomy or hemithyroidectomy (HT) with prophylactic unilateral (ipsilateral) CND was performed. Total thyroidectomy was performed when more than TI-RADS 3 types of thyroid nodules were identified in the contralateral lobe. CN suspension was used or not before surgery. (3) The central lymph nodes were independently evaluated as negative by two professional ultrasound physicians. The exclusion criteria were as follows: (1) Largest tumor diameter > 2 cm. (2) Patients with PTC coexisting with Hashimoto’s thyroiditis often have more lymph nodes in the central neck compartment. To understand the true effect of CNs in increasing the number of harvested lymph nodes, patients with Hashimoto’s thyroiditis were excluded. (3) Bilateral nodes were diagnosed as PTC by FNA or pathological examination during the operation. In our center, bilateral LND will be performed under this circumstance. The main research subjects, including recurrent laryngeal nerve injury, lymph node harvest, and hypoparathyroidism, may be influenced by this surgical approach. (4) Patients who were not suitable for endoscopic surgery due to previous cervical surgery or other reasons. Fifty-four patients were finally included in the CN group, while 72 patients were included in the control group. Medical records, including intact parathyroid hormone (iPTH) results and histopathological examination of lymph nodes, were reviewed retrospectively. The follow-up times were 6 to 42 months.

### CN suspension injection

**Fig. 1:**
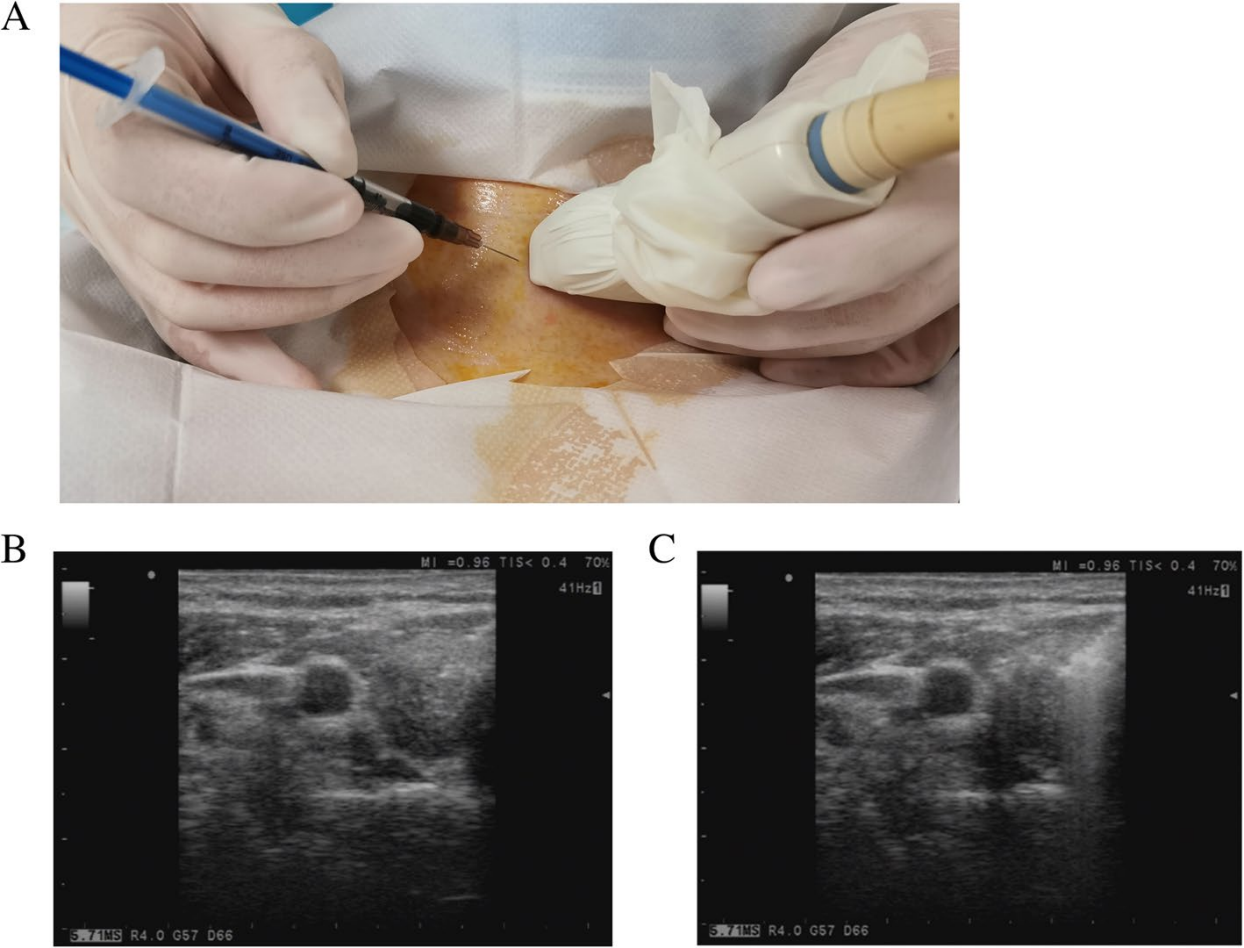
Preoperative ultrasound-guided CN suspension injection. **A** CN suspension was injected by using a 1-ml syringe. **B** Before the CN suspension injection, the needle can be seen under ultrasound guidance. **C** After the CN suspension injection, a high-echo area was formed in the thyroid gland

All of the patients underwent preoperative (the day before surgery) ultrasound-guided CN suspension injection. A 0.2-mL suspension was injected into the normal thyroid tissue (the lobe with the primary tumor) beneath the thyroid capsule (Fig. 1A, B).

### Surgical procedure

**Fig. 2:**
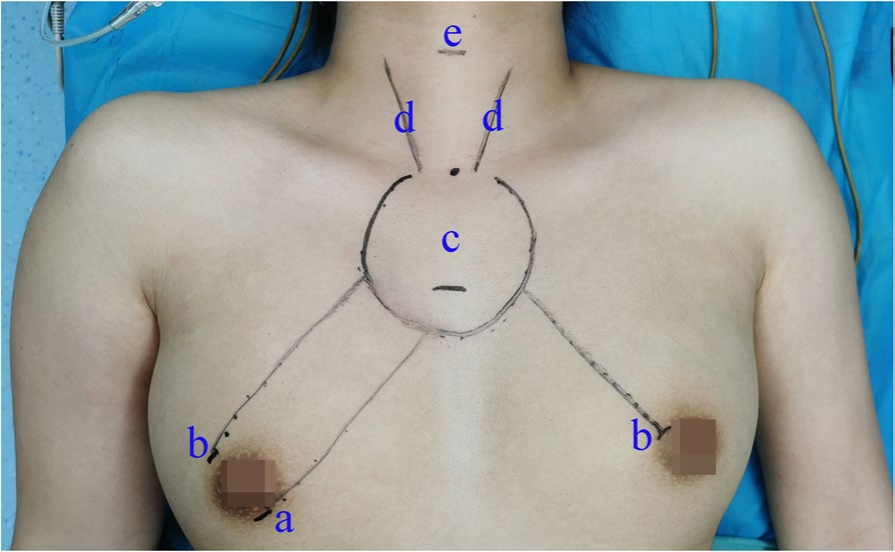
Incisions and working space. (a) One 10-mm incision for endoscopy. (b) Two 5-mm incisions for operating instruments. (c) Working space, freed flap area in the chest wall. (d) Anterior edge of sternocleidomastoid muscle. (e) Body symbol of annular cartilage

Endoscopic thyroidectomy and pCND were performed as described previously [13]. In brief, after general anesthesia, epinephrine was used for spacing creation. One 10 mm incision at the right areola for laparoscopy and two 5-mm incisions at the bilateral areola for operating instruments were made for trocar location (Fig. 2). The working space was inflated with CO_2_ gas at a pressure of 6–8 mmHg. Ultrasonic coagulation devices were used to divide the vessels. The isthmus of the thyroid gland, trachea, and common carotid artery was exposed in that order. The middle thyroid vein, inferior thyroid arteries, and veins were isolated. The RLN was exposed and dissected in the caudad to cephalad direction, and the thyroid lobe was subsequently removed. Unilateral CND was performed covering the prelaryngeal, pretracheal, and ipsilateral paratracheal areas.

### Statistical analysis

Continuous variables are shown as the mean ± standard deviation. Statistical analysis was performed using Student’s *t* test, Fisher’s exact test, and the chi-square test with SPSS 23.0 software. A *P* value < 0.05 was considered statistically significant.

## Results

### General information

The demographic and clinicopathological characteristics of the CN and control groups are compared in Table 1. All endoscopic thyroidectomies were successfully performed. Age, sex, and tumor size were similar in the two groups. Fifteen patients underwent total thyroidectomy and 39 lobectomies in the CN group, while 20 patients underwent total thyroidectomy and 52 lobectomies in the control group. All of the patients underwent prophylactic unilateral (ipsilateral) CND. No significant difference was observed for operative time, transient postoperative hoarseness, hematoma/seroma, or paresthesia of the chest wall between the two groups. There were only 1 and 2 cases of permanent vocal fold paralysis in the CN group and the control group, respectively. Both groups showed no recurrence in the thyroid gland or central and lateral neck compartments during the follow-up times.

**Table 1:** Demographic data and surgical outcomes of patients undergoing endoscopic thyroidectomy with CN group versus control group

Variables	CN	Control	*p* value
Age, mean ± SD	34.7 ± 9.8	35.0 ± 9.1	0.834
Gender			
Male	2	2	0.581
Female	52	70	
Tumor size (mm, mean ± SD)	8.7 ± 2.8	8.8 ± 2.6	0.944
Extent of surgery			
TT+ CND	15	20	0.562
HT+ CND	39	52	
Operation time (mins, mean ± SD)			
TT+ CND	129.3 ± 16.7	125.0 ± 23.7	0.550
LT+ CND	88.0 ± 10.5	87.1 ± 11.5	0.708
Postoperative hoarseness cases			
Transient	3	4	0.650
Permanent	1	2	0.607
Hypoparathyroidism			
Transient	5	7	0.581
Permanent	0	0	-
PTH levels (day 1, pg/ml)			
Without TH	30.0 ± 10.0	29.3 ± 9.4	0.716
With TH	8.2 ± 2.6	8.7 ±3.2	0.774
PTH levels (day 3)			
Without TH	30.5 ± 10.4	29.5 ± 9.8	0.590
With TH	8.8 ± 1.6	9.4 ± 2.6	0.643
PTH levels (month 3)	34.3 ± 8.4	32.3 ± 7.1	0.141
Hematoma/seroma	1	0	0.432
Paresthesia of chest wall			
Within 3 months after surgery	10	13	0.578
Three months after surgery	0	1	0.429
Recurrence	0	0	-

*TT* total thyroidectomy, *HT* hemithyroidectomy, *CND* central neck dissection, *PGs* parathyroid glands, *TH* transient hypoparathyroidism

### Carbon nanoparticles do not improve the protective effect on the inferior parathyroid gland

**Fig. 3:**
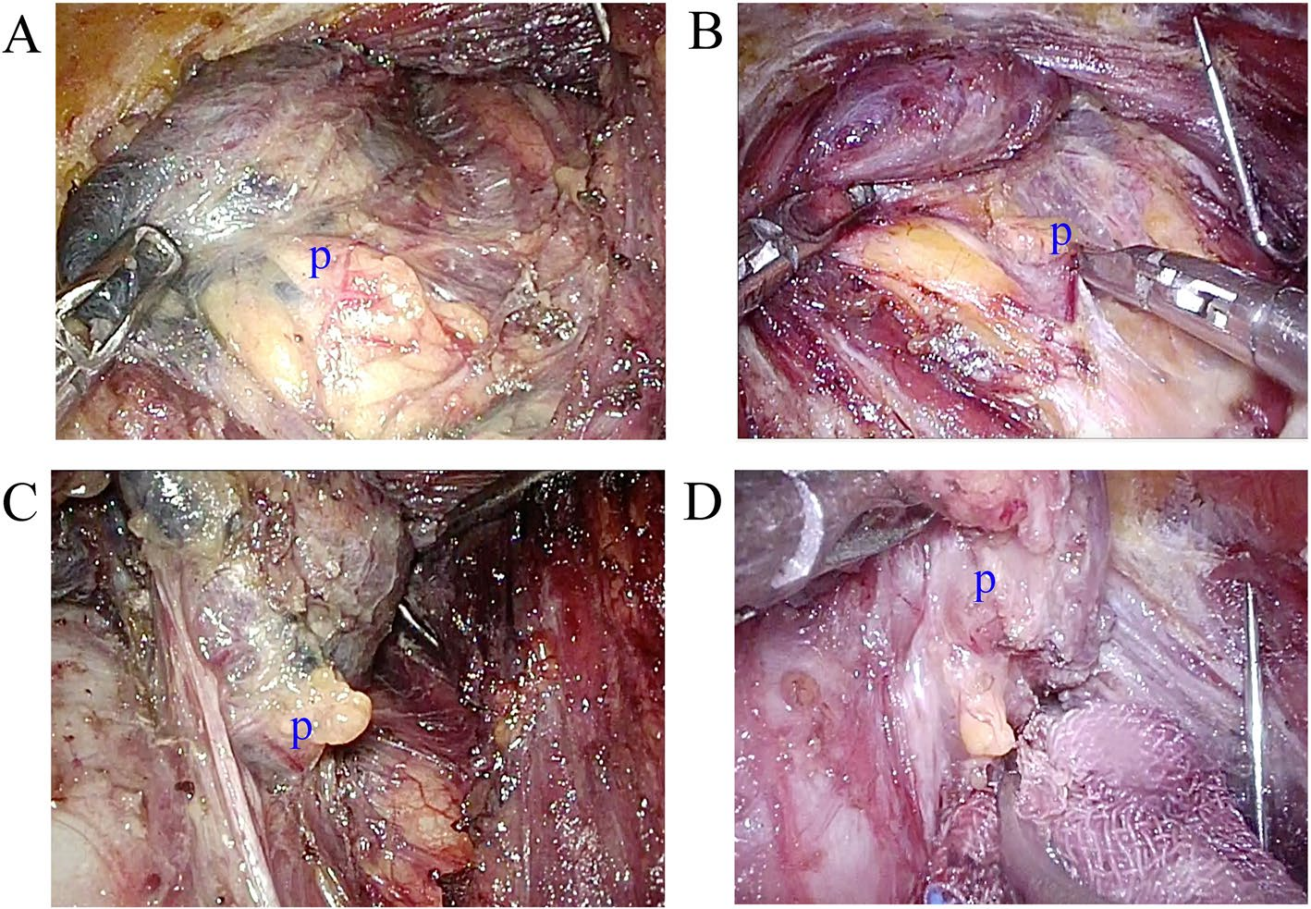
Exposure of the parathyroid glands. **A** Exposure of the inferior parathyroid gland with CN. **B** Exposure of the inferior parathyroid gland without CN. **C** Exposure of the superior parathyroid gland with CN. **D** Exposure of the superior parathyroid gland without CN

The number of transient hypoparathyroidisms (TH) in the control group and the CN group was 5 and 7, respectively. The serum PTH levels on postoperative days 1 and 3 were similar in both the CN and the control groups. The serum PTH levels dropped markedly in patients with TH on postoperative days 1 and 3. All of the PTH levels in patients with TH rose to normal levels within 3 months. Permanent hypoparathyroidism did not develop in either groups. Unilateral thyroid gland detachment is treated as a single superior and inferior parathyroid gland exposure. The position of the superior parathyroid gland is relatively fixed and can be identified easily during the surgical procedure. Only 5 and 8 of the superior parathyroid glands could not be clearly identified in the CN and control groups. The number of inferior parathyroid glands identified in situ or in the dissected central lymph tissues was 42 and 54 in the CN and control groups, respectively (Fig. 3, Table 2). This suggests that nanocarbon particles do not improve the protective effect on the parathyroid gland, especially the inferior glands.

**Table 2:** Identification of PGs and harvest of lymph nodes in CN group versus control group

Variables	CN	Control	*p* value
Numbers of identified PGs			
Superior PGs (not identified)	5/69	8/92	0.512
Inferior PGs	42/69	54/92	0.361
Numbers of harvested LNs (mean ± SD)			
LNs > 5mm	1.81 ± 1.30	2.03 ± 1.24	0.354
LNs < 5 mm	3.83 ± 1.50	2.00 ±1.18	< 0.001*
Total LNs	5.65 ± 2.62	4.06 ± 2.48	0.001*
Numbers of patients with metastatic LN	11	15	0.565
Numbers of metastatic LN**			
LN > 5mm	18	25	-
LN < 5 mm	3	2	-

*PGs* parathyroid glands, *CN* carbon nanoparticles, *LNs* lymph nodes

*p < 0.05

**In some cases, there were more than one metastatic LN

### Carbon nanoparticles only facilitate the harvest of lymph nodes with a diameter < 5 mm

**Fig. 4:**
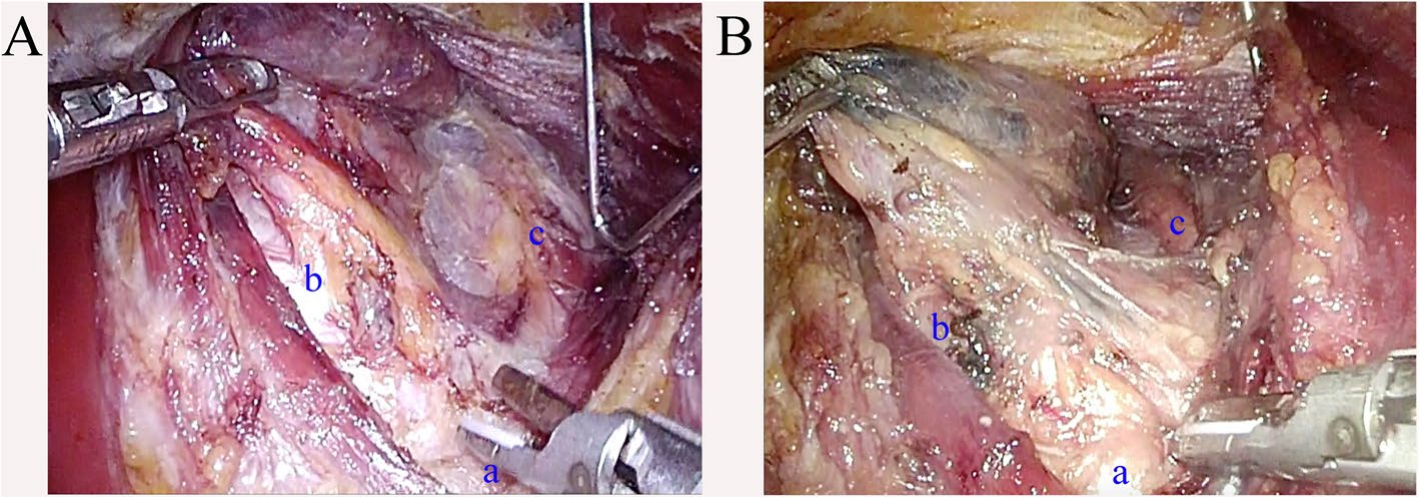
Central lymph nodes dissection. **A** CND without CN. **B** CND with CN. Locoregional lymph nodes were stained black. (a) Suprasternal fossae. (b) Trachea. (c) Common carotid artery

The lymph nodes were stained black after using CNs (Fig. 4). The mean number of lymph nodes removed by unilateral CND was 5.65 in the CN group and 4.06 in the control group. The number of cases with CLN metastasis was 11 and 15, respectively, which was relatively low. Statistical testing revealed that more lymph nodes under 5 mm were obtained in the CN group. Meanwhile, there was no difference in the harvested lymph nodes with a diameter greater than 5 mm. The metastasis rate of lymph nodes below 5 mm was extremely low, and only 5 lymph nodes were pathologically diagnosed as metastatic lymph nodes. This suggested that carbon nanoparticles facilitate lymph nodes harvesting only by identifying more lymph nodes with diameters < 5 mm. This may be partly attributed to the fact that black-stained lymph nodes are more likely to be detected by pathologists. Considering that carbon nanoparticles only facilitate harvesting lymph nodes with diameter < 5 mm with extremely low metastatic rates and have no influence on oncologic prognosis, it seems that there is no need to use lymph node tracers in prophylactic unilateral CND.

## Discussion

Compared with the traditional open thyroidectomy, endoscopic approaches are recommended to minimize the surgical extent for well-differentiated TC and to meet the growing demand for improved cosmesis [14]. Transaxillary, retroauricular, transoral, and bilateral axillo-breast approach (BABA) robotic approaches have been reported to achieve endoscopic thyroidectomy [15, 16]. Jiang et al. evaluated the safety and effectiveness of endoscopic thyroidectomy compared with conventional thyroidectomy in PTC by meta-analysis, showing that the two procedures were similar in safety, efficacy, and tumor recurrence [17]. In our study, all endoscopic thyroidectomies were successfully completed without conversion to open surgery. The complication rate was the same as that reported in open surgery. The rates of transient postoperative hoarseness and vocal fold paralysis were acceptable. During the follow-up period, no recurrence was detected in any of the patients. Endoscopic thyroid surgery through the total mammary areolas approach seems to be a feasible approach in selected patients with PTC.

CN has a good effect in dying lymph nodes black with a strong contrast from the surrounding tissues [18]. Several studies have confirmed the efficacy of CNs in lymph node dissection in thyroidectomy [18–21]. Li et al. reported that CNs could be used to help identify and remove PGs during parathyroidectomy [22]. Spartalis et al. conducted a systematic review to evaluate the benefits of CNs in thyroid surgical procedures and examine their role in lymph node tracing, parathyroid preservation, and recurrent laryngeal nerve protection. They revealed that carbon nanoparticles may improve both central and lateral neck dissection and enhance parathyroid gland identification and preservation [21]. However, Liu’s study demonstrated that although CNs helped identify PGs and LNs during surgery, the benefit brought by CNs in protecting the parathyroid glands and removing lymph nodes was not significant [20]. There have been no reports about the facilitative effect of CNs in protecting the parathyroid glands and harvesting the central lymph nodes in endoscopic thyroidectomy with pCND. In this study, we found that there was no difference in superior parathyroid gland exposure between the two groups due to its relatively fixed position. In a standard operating procedure, the mean number of inferior parathyroid glands identified in situ or in the dissected central lymph tissues is not influenced by the usage of CNs. This may be partly because the position of inferior parathyroid glands is variable. Inferior parathyroid glands in the surrounding region of the thyroid and central neck compartment can be visually recognized, with little help from CNs. However, parathyroid glands located in the thymus and in other uncommon positions cannot be seen regardless of the use of CNs. This suggests that nanocarbon particles do not improve the protective effect on the parathyroid gland, especially the inferior glands.

CN has been considered as a superior tracer for lymph nodes detection in multiple tumors, including TC. Although the true assistive role of CNs in lymph node harvest in thyroid surgery is somewhat controversial, the application of CNs is not limited to the initial surgical LND. For example, Chen et al. applied CNs to reveal drainage lymph nodes and to pick the lateral lymph nodes for pathological assessment in cN0 PTC patients, and modified radical lateral lymph node dissection was applied when metastasis presented [19]. CN injection was also reported to help perform CLND and identify PGs in reoperation, significantly lowering the incidence of transient hypoparathyroidism [23]. Meanwhile, preoperative CN injection is feasible and effective to localize the metastatic lymph nodes during reoperation [24]. Other researchers have reported that pCND is not associated with better short-term outcomes, and instead, it increases postoperative morbidity [25, 26]. In this study, the mean number of harvested lymph nodes in the CN group was greater than that in the control group. However, there was no difference in the harvested lymph nodes with a diameter greater than 5 mm. This suggests that carbon nanoparticles facilitate the lymph node harvesting only by identifying more lymph nodes with diameter < 5 mm. Considering that lymph nodes with diameter < 5 mm have extremely low metastatic rates and no influence on oncologic prognosis, it seems that the use of lymph node tracers in prophylactic unilateral CND is not urgently necessary.

## Conclusions

Carbon nanoparticles do not improve the protective effect on parathyroid glands, especially the inferior glands, in endoscopic thyroid surgery through the total mammary areolas approach. There is no need to use CNs to facilitate the lymph node harvest in endoscopic prophylactic unilateral CND.

## Data Availability

All data generated or analyzed during this study are included in this published article.
